# C-851-01. Advancements in Burn Size Assessment: A Systematic Review of Emerging Technologies

**DOI:** 10.1093/jbcr/irag033.053

**Published:** 2026-04-06

**Authors:** Amy Woods, Poh (Leslie) Tan, Christopher J Lewis, Tamara Mertz

**Affiliations:** Royal Victoria Infirmary, Newcastle, Newcastle upon Tyne, England; Northern Regional Burn Centre, Newcastle Upon Tyne, England; Northern Regional Burn Centre, Newcastle Upon Tyne, England; Royal Victoria Infirmary, Newcastle, Newcastle upon Tyne, England

## Abstract

**Introduction:**

Accurate and reproducible assessment of total body surface area (TBSA) in burns is vital for guiding fluid resuscitation, triage, referral, and overall clinical management. Traditional methods such as the Lund-Browder chart remain widely used but are limited by significant inter-rater variability and inaccuracies. These errors can result in adverse clinical outcomes, unnecessary referrals, and healthcare costs. This systematic review evaluates technologies developed to improve TBSA estimation, focusing on diagnostic accuracy, inter-rater reliability, and the quality of current evidence.

**Methods:**

A comprehensive search of EMBASE, MEDLINE (OVID), Web of Science, Scopus, PubMed and Cochrane Library was conducted for studies published in English between January 2010 and January 2025. Eligible studies assessed technological tools for TBSA estimation in human or artificial burns. Exclusion criteria included expert opinion, conference abstracts, and studies lacking quantitative outcomes. Primary outcomes were accuracy and inter-rater reliability. Secondary outcomes included speed and user experience. Risk of bias and applicability were appraised using QUADAS-2. PROSPERO registration: CRD42025632872.

**Results:**

Thirty-six studies met the inclusion criteria: 3D computer programs (n = 7), mobile applications (n = 11), 3D stereophotogrammetry (n = 8), and machine learning models (n = 10). Eight studies evaluating five 3D scanning systems demonstrated that stereophotogrammetry showed the highest accuracy (mean ICC = 0.988) and excellent inter-rater reliability (ICC = 0.989), with supporting evidence for adult and paediatric cohorts. 3D programs demonstrated good diagnostic performance and reduced variability but lacked the anatomical realism of stereophotogrammetry. Eleven of twelve mobile applications improved accuracy and consistency, particularly among non-specialists, and offered practical benefits in prehospital settings. Machine learning, though largely in experimental phases, demonstrated promising accuracy, with some models outperforming clinician estimates.

**Conclusions:**

Advances in imaging and digital tools show clear potential to improve burn size assessment, but their readiness for routine use varies. 3D stereophotogrammetry offers high precision within specialist centres, mobile applications enable rapid estimation in the field, and machine learning may support future automation.

**Applicability of Research to Practice:**

For clinicians, these technologies should be viewed as complementary rather than replacements for current standards. Mobile applications can aid rapid triage in prehospital or resource-limited settings, while 3D platforms provide support for surgical planning, education and documentation. Adoption into daily practice, however, must be guided by evidence from robust, real-world studies addressing usability, workflow integration, and measurable improvements in patient outcomes.

**Funding for the study:**

N/A.

